# Centiloid method evaluation for amyloid PET of subcortical vascular dementia

**DOI:** 10.1038/s41598-017-16236-1

**Published:** 2017-11-24

**Authors:** Hyuk Jin Yun, Seung Hwan Moon, Hee Jin Kim, Samuel N. Lockhart, Yearn Seong Choe, Kyung Han Lee, Duk L. Na, Jong-Min Lee, Sang Won Seo

**Affiliations:** 1 Department of Biomedical Engineering, Hanyang University, Seoul, 04763 Korea; 2 Fetal Neonatal Neuroimaging and Developmental Science Center, Division of Newborn Medicine, Boston Children’s Hospital, Harvard Medical School, Boston, 02115 MA USA; 3 Department of Nuclear Medicine, Sungkyunkwan University School of Medicine, Seoul, 06351 Korea; 4 Department of Neurology, Samsung Medical Center, Sungkyunkwan University School of Medicine, Seoul, 06351 Korea; 5 Neuroscience Center, Samsung Medical Center, Seoul, 06351 Korea; 6 Helen Wills Neuroscience Institute, University of California, Berkeley, 94720 CA USA; 7 Department of Internal Medicine, Division of Gerontology and Geriatric Medicine, Wake Forest School of Medicine, Winston-Salem, 27157 NC USA; 8 Department of Health Sciences and Technology, Sungkyunkwan University, Seoul, 06351 Korea; 9 Department of Clinical Research Design and Evaluation, SAIHST, Sungkyunkwan University, Seoul, 06351 Korea

## Abstract

Reference region selection is important for proper amyloid PET analysis, especially in subcortical vascular dementia (SVaD) patients. We investigated reference region differences between SVaD and Alzheimer’s disease (AD) using Centiloid scores. In 57 [C-11] Pittsburgh compound B (PiB) positive (+) AD and 23 PiB (+) SVaD patients, we assessed standardized PiB uptake and Centiloid scores in disease-specific cortical regions, with several reference regions: cerebellar gray (CG), whole cerebellum (WC), WC with brainstem (WC + B), pons, and white matter (WM). We calculated disease group differences from young controls (YC) and YC variance according to reference region. SVaD patients showed large effect sizes (Cohen’s *d* > 0.8) using all reference regions. WM and pons showed larger YC variances than other regions. Findings were similar for AD patients. CG, WC, and WC + B, but not WM or pons, are reliable reference regions for amyloid imaging analysis in SVaD.

## Introduction

The use of amyloid PET scans for quantitative measurement of amyloid-beta deposition has grown in recent years. As a result, the development and standardization of methods for amyloid-PET data analysis, particularly methods applicable across centers, has increasingly interested clinicians and investigators^1–3^. The Centiloid project, an effort to standardize quantitative amyloid-PET plaque estimation across centers, is one such development. The ratio of target to reference region is an effective method for calculating amyloid load, with sufficient discriminatory power between Alzheimer’s disease (AD) and matched controls^2,4^. This method is easy, useful, has obvious merit for clinical use as it requires no arterial blood sampling^2^, and has been adopted widely for semi-quantification of amyloid load. In probable AD, target to reference ratio is usually large and variance of the ratio matters little clinically^2^. However, there are sometimes subjects with borderline amyloid PET tracer uptake. Aβ deposition occurs on a continuum; at present there is no clear a priori way to separate individuals who have Aβ in the brain from those who do not. Therefore, small differences in quantification result according to the reference region used can present an issue especially in subjects with borderline uptake. Furthermore, when target-to-reference ratios are converted to the Centiloid scale, the values are increased, as the dynamic range of the Centiloid scale is greater than that of the target-to-reference ratio; consequently, choosing an inappropriate reference region may exaggerate errors in the Centiloid procedure especially in patients with borderline amyloid burden.

Cerebrovascular disease (CVD) and amyloid burden are the most frequent pathologies in cognitively impaired subjects^5^, and these two distinct pathologies present concurrently at a high rate. Mixed pathology is present in approximately half of all clinically diagnosed AD cases^6–9^, even in clinical trials with participants extensively screened for pure AD^10^. Conditions such as subcortical vascular dementia (SVaD), which exhibit both CVD and amyloid pathology, also require amyloid quantification; however, reference region selection in SVaD presents additional difficulties because pathology may occur within and impact measurement of certain reference regions.

According to Thal amyloid phase, pons and cerebellum are among the latest regions presenting amyloid pathology in AD^11^. The cerebellum has been used as a reference region in most previous studies using [C-11] Pittsburgh compound B (PiB) PET scans^2,4,12^. However, there is concern that cerebellum may not be suitable as a reference region for analyses of amyloid burden in conditions other than late-onset AD, as cerebellar amyloid deposits may be present in cerebral amyloid angiopathy (CAA)^13^, prion diseases^14^, and genetic AD^15,16^. Therefore, alternative reference regions such as pons have been evaluated for reliability^4^. Edison and colleagues reported that the pons is a reliable reference region for analysis in [C-11] PiB studies where cerebellum is not an appropriate reference region^4^. A recent study also suggested that WM as a reference region improved discrimination between clinically-defined groups^17^. However, the appropriateness of different reference regions for assessing amyloid uptake in patients with SVaD has not yet been investigated.

In this study, we investigated the use of different reference regions in analyzing amyloid uptake in SVaD patients. Given that SVaD patients affect more severe white matter (WM), brainstem and cerebellum than AD patients^18–20^, we hypothesized that there might be differences between SVaD and AD in the appropriateness of different reference regions. For testing the hypothesis, we used five reference regions cerebellar gray matter (CG), whole cerebellum (WC), WC with brainstem (WC + B), pons, and WM which have been frequently used in previous studies.

## Results

### Participant demographics

Table 1 shows demographic information of participants in this study. There were no differences in gender proportion between PiB (−) old control (OC), PiB (+) AD, and PiB (+) SVaD groups. However, ANOVA (*F* = 14.2, p < 0.001) and post-hoc tests indicated the PiB (+) AD group was younger than the PiB (−) OC (t = 3.4 and p = 0.001) and PiB (+) SVaD (t = 4.3 and p < 0.001) groups.

**Table 1:** Demographics and clinical findings of subjects.

	PiB(+) AD	PiB(+) SVaD	PiB(−) OC	PiB(−) YC
*n*	57	23	14	34
Gender (M/F)	21/36	7/16	9/5	N/A
Age (mean ± SD)	69.0 ± 8.3	78.0 ± 6.8^*^	78.0 ± 5.0^*^	31.5 ± 6.3^*^

PiB:^11^C-Pittsburgh compound B; AD: Alzheimer’s disease; SVaD: subcortical vascular dementia; OC: old control; YC: young control; OC: data from Alzheimer’s Disease Neuroimaging Initiative (ADNI) for generating cortical target region; YC: data used in Klunk *et al*.^1^ for statistical test (the detail demographics of age and gender were not available); *n*: the number of subjects; M: male; F: female; and SD: standard deviation; Asterisk(*): significant difference from group of PIB(+) AD.

### Global cortical target (CTX) ROIs in PiB (+) AD and PiB (+) SVaD

**Figure 1:**
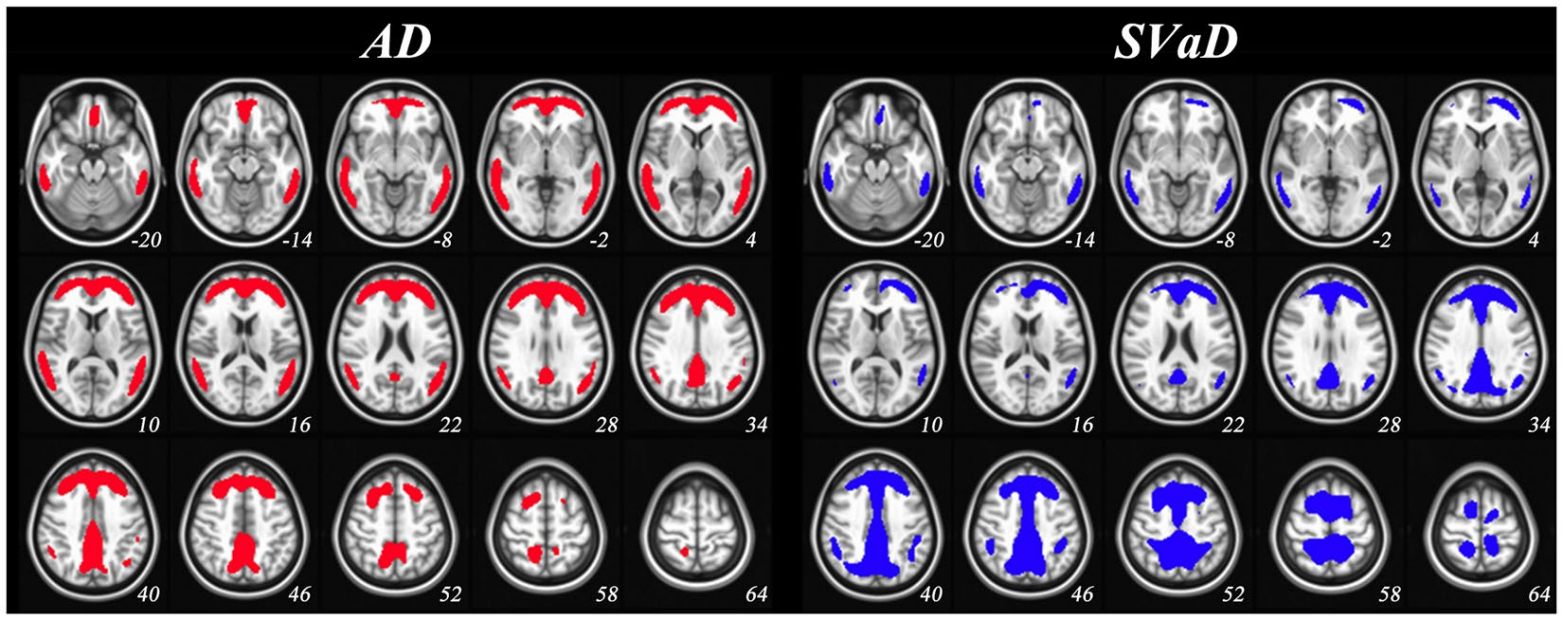
Disease-specific cortical target regions. AD: Alzheimer’s disease; SVaD: subcortical vascular dementia. Colored areas indicate voxels by percent threshold (upper 3% intensity) of group-averaged difference between old control and patient groups, which was defined as disease-specific cortical target regions. The regions are mapped on MNI 152 template with the coordinates of the z-axis (numbers under each slice) to the MNI space.

Figure 1 shows disease-specific CTX ROIs representing large differences in group-averaged PiB images between OC and patients (see the section of “***Disease-specific CTX ROI***”). SUVr of every voxel in the ROIs is significantly higher in patients (see Supplementary Fig. S1). Both CTX_AD_ and CTX_SVaD_ were mostly located in frontal, temporal and parietal regions. When evaluating proportions by lobe, CTX_SVaD_ showed relatively larger proportions in parietal and lower proportions in temporal regions than CTX_AD_ (Table 2).

**Table 2:** The proportion of CTX regions.

	Frontal lobe	Parietal lobe	Temporal lobe	Occipital lobe	Total
**CTX**_**AD**_	56.71%	17.89%	23.04%	2.36%	100%
**CTX**_**SVaD**_	54.51%	33.86%	10.78%	0.85%	100%

CTX: cortical target; AD: Alzheimer’s disease; SVaD: subcortical vascular dementia.

### Effect size of Centiloid (CL) values between disease groups and young control (YC) according to reference ROIs

The differences of CL between disease groups and YC are represented as effect sizes (Table 3). The effect sizes were large enough (Cohen’s *d* > 0.8) to show consistent group differences in all reference ROIs. The highest effect size of differences between PiB (+) AD and YC was generated using WM ROI (5.543) followed by pons (4.363), WC + B (4.200), WC (4.043) and CG (3.698). The highest effect size of differences between PiB (+) SVaD and YC was generated using WM ROI (4.721) followed by WC + B (3.957), WC (3.928), CG (3.851) and pons (3.615).

**Table 3:** Summary statistics of SUVr and Centiloid scores in disease-specific cortical target regions from each of five reference regions

		SUVr						Centiloid score					Bartlett’s statistic
			CG	WC	WC + B	Pons	WM	CG	WC	WC + B	Pons	WM	
AD	Patient	Mean	2.457	2.073	1.973	1.571	1.219	100.000	100.000	100.000	100.000	100.000	
		SD	0.445	0.335	0.307	0.234	0.107	33.643	30.708	29.525	28.065	21.001	12.685^*^
	YC	Mean	1.135	0.981	0.933	0.739	0.709	0.000	0.000	0.000	0.000	0.000	
		SD	0.054	0.049	0.049	0.062	0.054	4.097	4.500	4.743	7.393	10.616	45.891^*^
	Effect size		3.698	4.043	4.200	4.363	5.543						
SVaD	Patient	Mean	2.368	1.997	1.896	1.496	1.220	100.000	100.000	100.000	100.000	100.000	
		SD	0.476	0.383	0.360	0.305	0.147	39.715	38.856	38.507	41.535	30.042	2.531
	YC	Mean	1.169	1.011	0.961	0.761	0.730	0.000	0.000	0.000	0.000	0.000	
		SD	0.058	0.051	0.051	0.062	0.053	4.847	5.212	5.461	8.428	10.732	33.244^*^
	Effect size		3.851	3.928	3.957	3.615	4.721						

AD: Alzheimer’s disease; SVaD: subcortical vascular dementia; SUVr: standardized uptake value ratio; CG: cerebellar gray; WC: whole cerebellum; B: brainstem; WM: white matter; SD: standard deviation; YC: young control; Asterisk(*): significant difference based on Bartlett’s statistics among five reference ROIs; Effect sizes were calculated using Cohen’s *d* with pooled standard deviation (See Eq. 2).

### Variance of CL values according to reference ROIs in YC group

**Figure 2:**
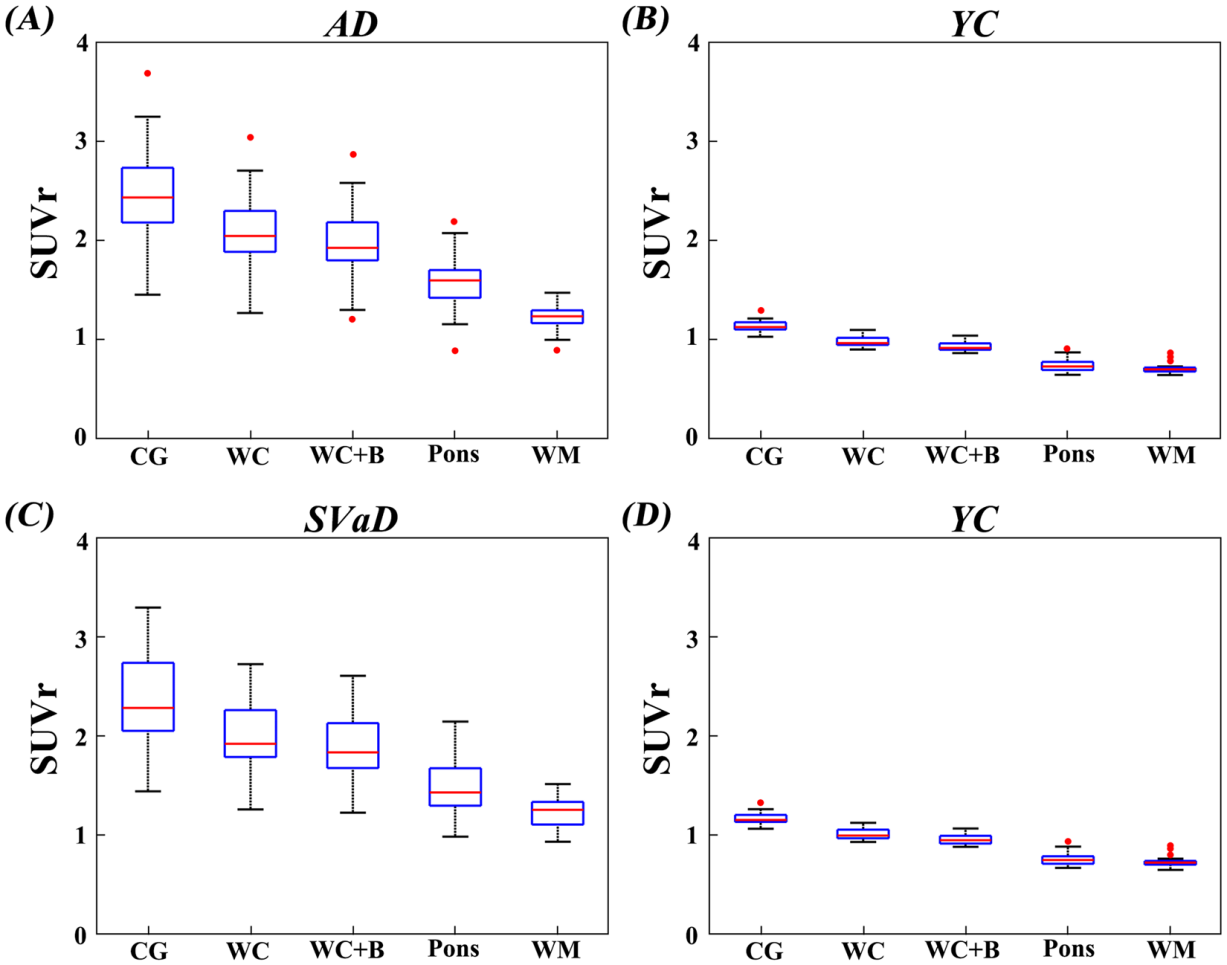
Post hoc variance tests of SUVr. Box plots display the SUVr distribution obtained by each reference. SUVr: standardized uptake value ratio; AD: Alzheimer’s disease; SVaD: subcortical vascular dementia; YC: young control; CG: cerebellar gray; WC: whole cerebellum; WC + B: WC with brainstem; WM: white matter. On each plot, the red line indicates the median (Q2), the bottom and top edges of the box represent lower quartile (Q1) and upper quartile (Q3). The outliers (red dots) are the values which falls outside of the lower/upper fences between 1.5 times the interquartile range (IQR, Q3–Q1) from Q1 and Q3, respectively. The black horizontal lines display minimum and maximum values in the fences.

**Figure 3:**
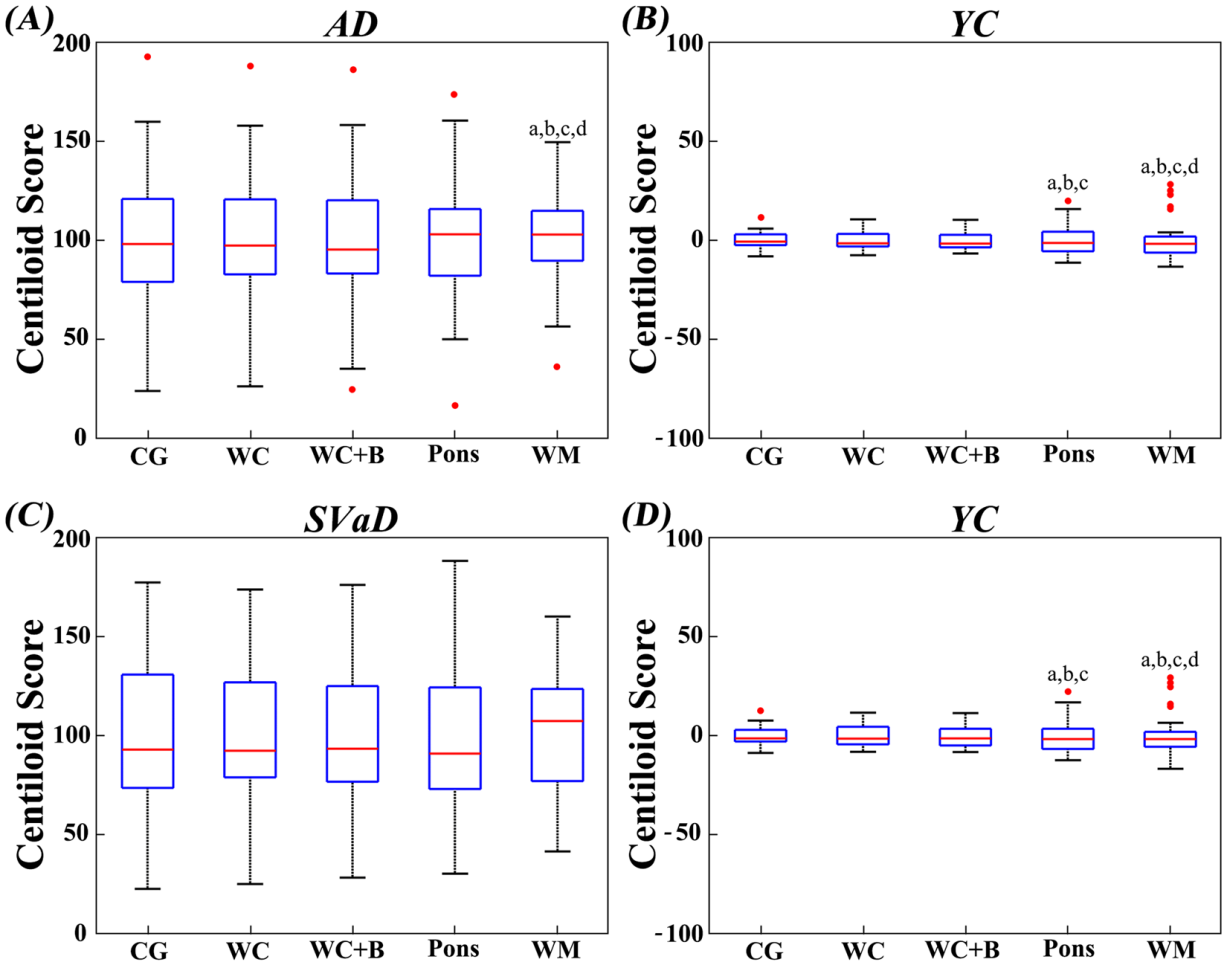
Post hoc variance tests of Centiloid score. Box plots display the variances and differences of Centiloid scores within group. Lowercase letters indicate significant differences from CG (**A**), WC (**B**), WC + B (**C**), and pons (**D**). AD: Alzheimer’s disease; SVaD: subcortical vascular dementia; YC: young control; CG: cerebellar gray; WC: whole cerebellum; WC + B: WC with brainstem; WM: white matter. On each plot, the red line indicates the median (Q2), the bottom and top edges of the box represent lower quartile (Q1) and upper quartile (Q3). The outliers (red dots) are the values which falls outside of the lower/upper fences between 1.5 times the interquartile range (IQR, Q3–Q1) from Q1 and Q3, respectively. The black horizontal lines display minimum and maximum values in the fences.

The results of assessing variance of CL values are shown in Table 3. The Bartlett’s statistics for equal variance indicated that there was within-group variability for all groups except SVaD patients; the results of *post hoc* tests are shown in Figs 2 and 3. The largest variances of CL values in YC were found using WM as reference region, followed in order by pons, WC + B, WC and CG (Table 3). Compared to other ROIs, WM and pons ROIs showed greater variance of CL values.

## Discussion

In this study, we translated our PiB PET data into the Centiloid scale as described previously^1^, and tested multiple reference regions for evaluating amyloid-beta deposits in cortical target regions, with a focus on patients with PiB (+) SVaD. We found that there were large magnitudes of effect sizes for differences between SVaD and YC regardless of reference ROI. However, using WM or pons ROI as a reference region showed larger variances in YC than using other ROI regions. In PiB (+) AD patients, the results were similar. Taken together, our findings show that CG, WC, and WC + B could be used as reference regions for amyloid imaging analysis in patients with PiB (+) SVaD, which are the same for AD patients.

We found that CG, WC or WC + B were reliable as reference regions for amyloid PET analyses in SVaD patients. These regions are commonly used as reference for amyloid PET analyses in AD patients because they are among the last regions where amyloid pathology is known to occur^2,4,12^, and are classified as Thal phase 5^11^. Although some studies have raised the possibility of requiring different reference regions for specific diseases^4,14–16^, our findings demonstrate that appropriate reference regions for SVaD patients are the same as those for AD.

In this study, WM showed higher variability of Centiloid-scaled values in YC group than other reference regions. [C-11] PiB binding to WM is mainly non-saturable and non-specific^21^, but PiB retention has been found to be reduced in WMH regions compared to normal appearing WM regions^22^. Some have raised concerns that using a WM-containing reference region for analysis of amyloid imaging data could be problematic in a population with high WMH^22^. Consistent with this, our findings suggest that the quantification of amyloid deposits based on the ratio of target to WM may be an inappropriate approach in SVaD patients.

We also found that pons showed higher variability than other reference regions of Centiloid-scaled values in YC. This may appear inconsistent with a recent amyloid imaging study demonstrating the value of the pons as a reference region^4^. The discrepancy may be partly explained by different analysis methods, as well as by different study subjects. Unlike the previous study, patients with genetic AD and prion disease were excluded in our study. However, our findings are consistent with a previous Centiloid study showing pons with higher variability and lower effect size than other reference regions^1^. In addition, the inherent variability of normalization processes could affect the results of our study. For example, poor performance of normalization process in the pons can occur frequently, due to its small size and the fact that the SPM normalization algorithm seems to handle brainstem structures less well than cortical structures^1^, and could influence the [C-11] PiB processing and results.

One of the important findings of our study is that CG, WC, and WC + B, but not pons nor WM, are appropriate reference regions in both AD and SVaD. However, several limitations must be noted. First, our conclusions that WM and pons are not reliable reference regions, while CG, WC, and WC + B are suitable for analysis in the scaled data set, is not based on neuropathological studies. The precise performance of these reference regions will require confirmation by further neuropathological studies. Second, other pathologies such as prion disease, tau pathology, or hippocampal sclerosis are not considered because we did not perform a pathologic study. Third, due to the small number of SVaD patients used in this study, it will be necessary to replicate our results with larger samples in further study. Fourth, selecting a disease-specific CTX ROI as well as an appropriate reference region is important for calculating amyloid uptake. We therefore chose the disease-specific CTX ROI in the SVaD and AD groups, separately. Finally, we did not perform correction of PiB retention for brain atrophy, which might have effects on quantification.

Nevertheless, this study demonstrates that CG, WC, and WC + B are reliable reference regions for amyloid PET analysis in patients with SVaD, suggesting that amyloid burden in SVaD patients might be analyzed using the same reference regions as AD.

## Methods

### Participants

We selected 57 patients with PiB (+) AD and 23 patients with PiB (+) SVaD according to clinical diagnosis and PiB-PET status (see below for PiB-PET methods). PiB(+) AD patients were those who (1) met probable AD dementia criteria proposed by the National Institute of Neurological and Communicative Disorders and Stroke and the AD and Related Disorders Association^23^; (2) had minimal WM hyperintensities (WMH; periventricular WMH < 5 mm and deep WMH < 5 mm); (3) and had significant amyloid burden (PiB standardized uptake value ratio [SUVr] ≥ 1.5) as measured by PiB-PET. Patients with PiB (+) SVaD were those who (1) met the diagnostic criteria for vascular dementia as determined by the Diagnostic and Statistical Manual of Mental Disorders–Fourth Edition (DSM-IV) and also fulfilled the imaging criteria for SVaD proposed by Erkinjuntti *et al*.^24^; (2) showed a subcortical vascular feature defined as both a focal neurological symptom/sign and significant ischemia on MRI; (3) had significant ischemia on MRI, defined as a cap or band ≥ 10 mm and a deep WM lesion ≥ 25 mm, as modified from Fazekas ischemia criteria^25^; and (4) had significant amyloid burden (PiB SUVR ≥ 1.5)^26^.

Patients were evaluated by clinical interview and neurological and neuropsychological examinations as previously described^27^. All patients underwent laboratory tests including complete blood count, blood chemistry, vitamin B_12_/folate, syphilis serology, and thyroid function tests. Brain MRI confirmed the absence of structural lesions including territorial cerebral infarction, brain tumors, hippocampal sclerosis, and vascular malformation.

### Ethics statement

This study protocol was approved by the Institutional Review Board of Samsung Medical Center. We obtained written consent from each participant and all methods were carried out in accordance with the approved guidelines.

### MR and [C-11] PiB-PET imaging techniques

We acquired standardized three-dimensional T1 turbo field echo images from all participants at Samsung Medical Center, using the same 3.0 T MRI scanner (Philips Achieva; Philips Healthcare, Andover, MA), using the following parameters: sagittal slice thickness, 1.0 mm, over contiguous slices with 50% overlap; no gap; repetition time (TR) of 9.9 msec; echo time (TE) of 4.6 msec; flip angle of 8°, and matrix size of 240 × 240 pixels, reconstructed to 480 × 480 over a field of view (FOV) of 240 mm.

All patients underwent [C-11] PiB-PET imaging at Samsung Medical Center or Asan Medical Center (Seoul, Korea) with identical settings using a Discovery STe PET/CT scanner (GE Medical Systems, Milwaukee, WI, USA). [C-11] PiB-PET scanning was performed in 3-dimensional scanning mode examining 35 slices of 4.25-mm thickness spanning the entire brain. [C-11] PiB was injected into an antecubital vein as a bolus with a mean dose of 420 MBq (range 259–550 MBq). CT scans were performed for attenuation correction 60 minutes after injection. A 30-minute emission static PET scan was then initiated. The specific radioactivity of [C-11] PiB at time of administration was more than 1,500 Ci/mmol for patients and the radiochemical yield was more than 35%. The radiochemical purity of the tracer was more than 95% in all PET studies.

### Image processing

We employed image processing described in the Centiloid paper^1^, and replicated the Centiloid procedure to validate methodological consistency (see Supplementary information).

### Disease-specific CTX ROI

In Klunk *et al*.^1^, the CTX ROI was defined using a data-driven mask thresholded at 1.05 SUVr (using the WC reference ROI) difference between group-averaged PiB images of 19 AD subjects and 25 age-matched older controls. The threshold was designed to avoid WM regions and to minimize the number of separate clusters in the CTX ROI, and small holes and isolated voxels were manually edited. Although the typical amyloid binding regions reported in previous studies were included in the published CTX ROI, there were two reasons to generate a new CTX ROI. First, the published CTX ROI was designed specifically for AD patients, and given different progression patterns between SVaD and AD^26^, it was reasonable to make a new CTX ROI for SVaD patients. The second reason necessitating a new CTX ROI was the use of a numerical SUVr threshold. While amyloid deposition was well represented using a CTX ROI 1.05 SUVr unit difference in the dataset of Klunk *et al*.^1^, this value has not been validated in other datasets. CTX ROI values may be highly variable across cohorts, especially in AD patients with lower severity. Therefore, new CTX ROIs for SVaD and AD patients (CTX_AD_ and CTX_SVaD_ ROIs) were generated (using WC as reference) using data from the 57 PiB (+) AD and 23 PiB (+) SVaD patients, as well as control data from the Alzheimer’s Disease Neuroimaging Initiative (ADNI). When creating disease-specific CTX ROIs, we compared disease group and 14 PiB (−) OC subjects to find regions of high amyloid accumulation in elderly. As described above, individual images were transformed to MNI-152 template space and SUVr values derived. The average OC image was (separately) subtracted from averaged AD and SVaD images and spatially smoothed with a 5 mm full width at half maximum kernel. To extract CTX_AD_ and CTX_SVaD_ ROIs, we applied a percent threshold (upper 3% intensity) to smoothed images to retain a similar number of voxels as the published CTX ROI^1^. These disease-specific CTX ROIs are shown in Fig. 1.

### Reference ROIs

The four data-driven ROIs (CG, WC, WC + B and pons) in Klunk *et al*.^1^ have been used as reference ROIs for quantifying of amyloid retention in numerous previous studies^28–33^, because no significant binding differences between AD and OC are observed. WM has also been evaluated as a reference ROI in recent [C-11] PiB-PET studies^34^, as those studies reveal comparable WM amyloid burden between diagnostic groups and low inter-subject variability. Therefore, we included WM as a candidate reference region. To generate the WM ROI, the WM probability map on template of SPM8 was thresholded at 0.7. This threshold provided a WM region that avoided contributions from CSF and amyloid retention in gray matter.

### Statistical analysis

Individual SUVr values were calculated for normalized PiB-PET using five different reference ROIs (including WM), and CTX_AD_ and CTX_SVaD._ SUVr values were obtained by ratio with each of the reference ROIs. CL values for individual subjects in AD group were computed by comparing to YC group which are considered not to have any brain amyloid pathology, and it defined as follows:

1 $${\rm{CL}}=100\times (SUV{r}_{IND\ast }-SUV{r}_{YC-0\ast })/(SUV{r}_{AD-100\ast }-SUV{r}_{YC-0\ast })\,$$

Where $SUV{r}_{IND\ast }$ represents the individual SUVr values of all YC-0 and AD-100 subjects, and $SUV{r}_{YC-0\ast }$ and $SUV{r}_{AD-100\ast }$ represent each group’s mean values. For SVaD group, the term of $SUV{r}_{AD-100\ast }$ was replaced as $SUV{r}_{SVaD-100\ast }$.

To describe the dissimilar amyloid binding patterns between CTX_AD_ and CTX_SVaD_ ROIs, we measured regional volumetric proportion of CTX by dividing into frontal, parietal, temporal, occipital lobe and other regions.

Selection of the standard reference was based on the effect size of the group differences between patients and YC and on the variance of reference ROIs. Effect size for each group was evaluated with Cohen’s *d* with pooled standard deviation,

2 $${\rm{Effect}}\,{\rm{Size}}=({\mu }_{p}-{\mu }_{n})/\sqrt{({N}_{p}{{\sigma }_{p}}^{2}+{N}_{n}{{\sigma }_{n}}^{2})/({N}_{p}+{N}_{n}-2)}\,$$

where:

*μ*_*p*_ and *μ*_*n*_ mean the average of SUVr in each patient and YC group.

${\sigma }_{p}^{2}$ and ${\sigma }_{n}^{2}$ are the variance in each patient and YC group.

*N*_*p*_ and *N*_*n*_ are the number of subjects in each patient and YC group.

For variance tests, Bartlett’s statistics were employed for validating equal variances of reference ROIs in each clinical group^35^. *Post hoc* variance tests were also performed to investigate regional specificities for reference ROIs.

For quantitative analysis of lobar SUVr values among all groups, mean values of SUVr (using WC as reference) in each lobe were calculated.

## Electronic supplementary material

Supplementary Information
