# Supplementary material for: Centiloid method evaluation for amyloid PET of subcortical vascular dementia
Source: Sci Rep. 2017 Nov 24;7:16322. doi: 10.1038/s41598-017-16236-1 (PMC5701176; doi:10.1038/s41598-017-16236-1)

Supplementary Information

Centiloid method evaluation for amyloid PET of subcortical vascular dementia

Hyuk Jin Yun, PhD[1,2,+], Seung Hwan Moon, MD, PhD[3,+], Hee Jin Kim, MD, PhD[4,5], Samuel N. Lockhart, PhD[6,7], Yearn Seong Choe, PhD[3], Kyung Han Lee, MD, PhD[3], Duk L. Na, MD, PhD[4,5,8] Jong-Min Lee, PhD[1,*], Sang Won Seo, MD, PhD[4,5,8,9,*]

[1] Department of Biomedical Engineering, Hanyang University, Seoul 04763, Korea; [2] Fetal Neonatal Neuroimaging and Developmental Science Center, Division of Newborn Medicine, Boston Children's Hospital, Harvard Medical School, Boston 02115 MA, USA; [3] Department of Nuclear Medicine, [4] Department of Neurology, Samsung Medical Center, Sungkyunkwan University School of Medicine, Seoul 06351, Korea; [5]Neuroscience Center, Samsung Medical Center, Seoul 06351, Korea; [6]Helen Wills Neuroscience Institute, University of California, Berkeley, USA; [7]Department of Internal Medicine, Division of Gerontology and Geriatric Medicine, Wake Forest School of Medicine, Winston-Salem, NC 27157, USA; [8]Department of Health Sciences and Technology, [9]Department of Clinical Research Design and Evaluation, SAIHST, Sungkyunkwan University, Seoul 06351, Korea

[+]these authors contributed equally to this work
*corresponding authors

Correspondence and requests for materials should be addressed to SWS (sangwonseo@empal.com) or JML (ljm@hanyang.ac.kr)

Image processing

Structural MRI images were individually registered to the MNI template using normalized mutual information. PiB-PET images were aligned to the corresponding structural MRI and registered to apply transform matrices of structural MRI to MNI space[1]. Correction for intensity nonuniformity artifacts and spatial normalization of registered structural MRI were performed[2].

Replication of Centiloid standard method

Before processing our dataset, we replicated the Centiloid procedure to validate methodological consistency. All PiB-PET and structural MRI of 34 young controls (YC-0) and 45 AD patients (AD-100) analyzed in the Level-1 study[3] were downloaded from the GAAIN website (http://www.gaain.org). We also downloaded regions of interest (ROIs): four reference ROIs (cerebellar gray [CG], whole cerebellum [WC], whole cerebellum with brainstem [WC+B] and pons) and a global cortical target (CTX) ROI. We manually set the coordinates of the anterior commissure and the orientation of all downloaded images following a tutorial available on the GAAIN website, before aligning to the 2mm isotropic MNI-152 T1-weighted template. Reoriented structural MRI scans were processed by the same procedure. Individual PiB binding values were calculated from each of the ROIs for normalized PiB-PET images, and CTX standardized uptake value ratio (SUVr) values were obtained by dividing uptake value of CTX region by the means of each of the four reference ROIs.

We calibrated and validated our processes using both individual- and group-level criteria. First, percent difference between calculated and published[3] CTX SUVr values for each subject were tested for differences greater than 5%, and images which failed the individual-level criterion were modified to achieve greater accuracy in coordinates and

orientation. Second, the group-level criterion was assessed using two methods, percent difference and linear regression. Percent difference of mean values in each group was similar to the individual-level test, using a more restrictive (<2%) range of differences. For regression, individual CTX SUVr values were converted into Centiloid (CL) value using equation 2.2.1 in Klunk, et al. [3] as follows:

The slope and intercept of the regression line and R^2 were measured by simple linear regression. We then considered our process validated if the slope ranged 0.98 to 1.02, the absolute value of intercept was less than 2 and the R^2 was larger than 0.98 (see Supplementary Table S1). More detail on this process is available on the GAAIN website (http://www.gaain.org/).

Supplementary Table S1 1. Results of replication step

	**CG**	**WC**	**WC + B**	**Pons**
**Slope** (0.98 to 1.02)	0.9987	0.9978	0.9983	0.9984
**Intercept** (-2 to 2)	0.0746	0.1290	0.0962	0.0907
**R^2** (>0.98)	0.9987	0.9994	0.9994	0.9981
**Maximum individual error** (-5% to 5%)	3.4408	2.1275	2.3516	4.5661
**Mean error in AD** (-2% to 2%)	0.11	0.29	0.53	1.09
**Mean error in OC** (-2% to 2%)	0.16	0.33	0.45	0.18

CG: cerebellar gray; WC: whole cerebellum; B: brainstem; AD: Alzheimer's disease; OC: old control; Comparison of the Centiloid value yielded from this study with reported value in [3]. All the parameters of linear correlation analysis are within the limits set for all reference regions.

Figure S1. *t* statistics of voxels in the CTX ROI (calculated by t-test). All the voxels showed significantly high SUVr in patients ($p < 0.02$).

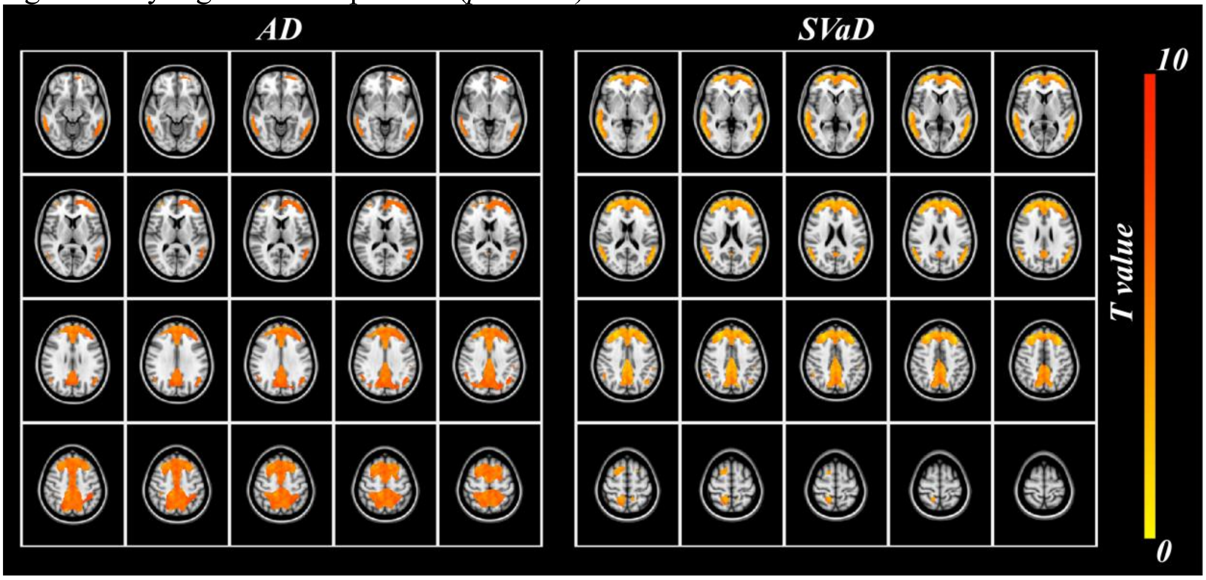

Supplement: Supplementary file 1 — Supplementary Information [file 41598_2017_16236_MOESM1_ESM.pdf]
